# Supramolecular polymer formation by a *de novo* hemoprotein with a synthetic diheme compound

**DOI:** 10.1002/2211-5463.12424

**Published:** 2018-05-11

**Authors:** Yasuhiro Isogai, Eisuke Takao, Ryuta Nakamura, Minoru Kato, Shigeki Kawabata

**Affiliations:** ^1^ Department of Pharmaceutical Engineering Toyama Prefectural University Imizu Japan; ^2^ Graduate School of Life Sciences Ritsumeikan University Kusatsu Japan; ^3^ Department of Liberal Arts and Sciences Toyama Prefectural University Imizu Japan

**Keywords:** AFM, *de novo* design, heme‐protein, supramolecular polymer

## Abstract

Proteins are attractive materials for supramolecular chemistry due to their multifunctionality and self‐organization ability. In this work, we synthesized a diheme compound, in which two iron‐protoporphyrin IX molecules are associated via a linker chain, and introduced it into a *de novo* designed four‐helix bundle protein with two heme‐binding sites. The protein gradually bound the diheme compound by *bis*‐histidyl ligation and formed supramolecular polymers. Polymer formation was observed by atomic force microscopy (AFM), which revealed the highly branched, dendritic forms of the fibrous architecture. The present results may open a pathway toward nanowire construction with *de novo* heme‐proteins.

**Abbreviations**
4HB: four‐helix bundle
AFM: atomic force microscopy

Supramolecular polymers are assembled from monomeric or oligomeric units through directional and repetitive noncovalent interactions between monomers 1. Many organic and metal compound‐containing biomolecules have been utilized for the self‐assembly of well‐organized architectures. In these molecular assemblies, each monomer typically has a pair of complementary chemical groups for binding other monomers. The monomer–monomer interactions, involving hydrogen bonds, metal coordination, and π–π interactions, are reversibly formed depending on the chemical environments, thus providing dynamic and functional features by responding to external stimuli. The degree of polymerization can be determined by the thermal equilibrium, that is, the monomer concentrations and the binding affinities between monomers.

Natural protein molecules exhibit various forms and functions, which are closely related to each other in both the monomeric and polymeric states. For example, animal myoglobins and hemoglobins have similar single‐chain folds, called the globin fold, but the former works for O_2_ storage in the monomeric form and the latter works for O_2_ transport in the tetrameric form 2. Many cytochromes with either single or multiple hemes are involved in electron transport with other redox proteins in respiration and photosynthesis 3. Virus capsid proteins fold into well‐defined monomeric structures and are assembled into highly organized supramolecular architectures 4.

*De novo* protein design was originally proposed by K. E. Drexler, as an approach to the development of molecular engineering 5. Since then, it has been a powerful tool to elucidate the principles of protein architecture. These studies have solved several relationships between the amino acid sequences and the 3D structures, functions, and physicochemical properties, regardless of the existing natural protein species. A four‐helix bundle (4HB), which is a naturally occurring protein structural motif, has been recursively adopted as the target structure for protein design 6, 7. Dutton and his colleagues designed and synthesized a series of heme‐binding 4HB proteins and revealed that the motif is suitable to explore the heme‐protein functions 8, 9, 10, 11, 12. We designed and synthesized artificial globins and *c*‐type cytochromes, and demonstrated that the redox functions are closely related to the protein folds and structural properties 13, 14, 15. The *de novo* design approach was also applied to recreate amyloid fibers and to elucidate the mechanisms of pathogenic protein self‐assembly 16, 17, 18. Self‐assembled small peptides have also been explored for biomedical and nanomaterial purposes 19, 20. Furthermore, protein engineering with natural heme‐ and metal‐binding proteins produced supramolecular polymers, with the aim of developing conductive nanowires 21, 22, 23, 24. However, *de novo* heme‐proteins remain unexplored for such purposes.

In this study, we have synthesized a novel diheme compound, in which two iron‐protoporphyrin IX (protoheme) molecules are associated with a linker chain, and introduced it into a *de novo* designed 4HB protein with two heme‐binding sites (Fig. 1A). The association of the heme compound with the protein resulted in the formation of nanofibrous scaffolds with highly branched, dendritic architectures.

**Figure 1:**
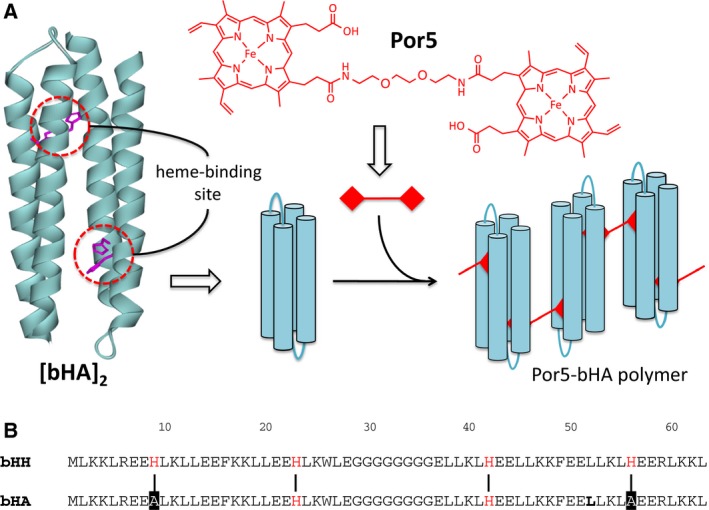
(A) Schematic representation of *de novo* heme‐protein polymer formation with the diheme compound. The 3D structure of the designed 4HB protein (left) was drawn by modification of the main chains for PDB code http://www.rcsb.org/pdb/search/structidSearch.do?structureId=1M3W 25. The His residues for ligation of the heme iron are colored magenta. (B) Amino acid sequence alignment between bHA and bHH 14. The His residues are positioned for ligation of the heme iron and indicated in red. The vertical lines between the sequences denote the positions assumed to be the heme axial ligands in both sequences. The two His residues at the positions 9 and 56 in the bHH sequence were replaced with Ala in bHA and are indicated by the black background.

## Materials and methods

### Design of the amino acid sequence

In the four‐helix bundle (4HB) polypeptide, bHH (*b*‐type bis‐His ligation), methionine was added to the N terminus of α*‐l‐*α, which was designed by Gibney *et al*. 26 to form the heme‐binding 4HB dimer ([α*‐l‐*α]_2_), to initiate the protein expression in *Escherichia coli* cells 14. The polypeptide, α*‐l‐*α or bHH, has two heme‐binding sites with bis‐His ligation, and thus, four heme‐binding sites are present in the 4HB unit, [α*‐l‐*α]_2_ or [bHH]_2_. The two His residues at the positions 9 and 56 in bHH were replaced with Ala in bHA to delete those heme‐binding sites and leave the other two heme‐binding sites formed by the two His residues at the positions 23 and 42 in 4HB (Fig. 1B).

### DNA construction

The artificial gene encoding bHA was synthesized by the polymerase chain reaction (PCR) from pbHH 14, a pRSET‐C vector (Invitrogen, Carlsbad, CA, USA) with the bHH gene cloned into the *Nde* I and *Hind* III restriction sites, as the template. The reaction was performed using the template DNA and the two primer DNA: 5′‐GGAGATATACATATGCTGAAGAAACTACGCGAAGAAGCGTTGAAGCTCCTAGAAGAG‐3′, encoding Ala‐9 in place of His‐9 in bHH and 5′‐GCCGGATCAAGCTTACAGCTTTTTCAAACGCTCCTCAGCCAGTTTCAGTAATTCCTC‐3′, encoding Ala‐56 in place of His‐56 in bHH with *Pyrobest* DNA polymerase (Takara Bio Inc., Otsu, Shiga, Japan). The resulting fragment was digested with *Nde*I and *Hin*dIII and cloned into pRSET‐C to generate pbHA. The synthetic oligonucleotides were purchased from (Eurofins Genomics Co. Ltd., Ebersberg, Germany).

### Protein expression and purification

The bHA gene in pRSET‐C was transformed into *E. coli* strain BL21 (DE3) and expressed in TB medium supplemented with 100 mg·L^−1^ ampicillin under the control of the T7 promoter using isopropyl 1‐thio‐β‐d‐galactopyranoside (IPTG). Cells were harvested by centrifugation, washed with TE buffer containing 10 mm Tris/HCl (pH 8.0) and 1 mm ethylenediaminetetraacetic acid (EDTA), and lysed by sonication in TE buffer supplemented with 0.3% (v/v) 2‐mercaptoethanol. After the addition of polyethyleneimine (PEI) to approximately 0.6%, the insoluble fractions containing DNA were removed by centrifugation. The supernatants were collected and dialyzed against 0.05% trifluoroacetic acid (TFA) overnight at 4 °C. After removal of the insoluble fractions by centrifugation, the supernatant was concentrated using an AMICON YM‐3 ultrafiltration membrane (Millipore, Bedford, MA, USA). The concentrated solution was centrifuged, and the supernatant was applied to a C18 reverse‐phase preparative HPLC column (Vydac 218TP1022, Grace, Deerfield, IL, USA). The apo‐bHA polypeptide was eluted with a 30–50% acetonitrile gradient in the presence of 0.05% TFA, using a Hitachi L‐6200 HPLC system. If necessary, the reverse‐phase chromatography was repeated to obtain a homogeneous polypeptide fraction with purity higher than 95%. The peak fraction containing bHA was collected and evaporated. The acidic polypeptide solution was neutralized by dialysis against a buffer solution containing 2 mm Tris/HCl (pH 8). The resultant solution was completely desalinated by further dialysis against water and lyophilized to obtain bHA powder. The covalent structure of the synthesized polypeptide was identified by SDS/PAGE and MALDI‐TOF mass spectrometry. The concentration of bHA was determined spectroscopically using ε_280_ = 5.8 mm ^−1^·cm^−1^ at pH 8.

### Synthesis of the diheme compound

**Figure 2:**
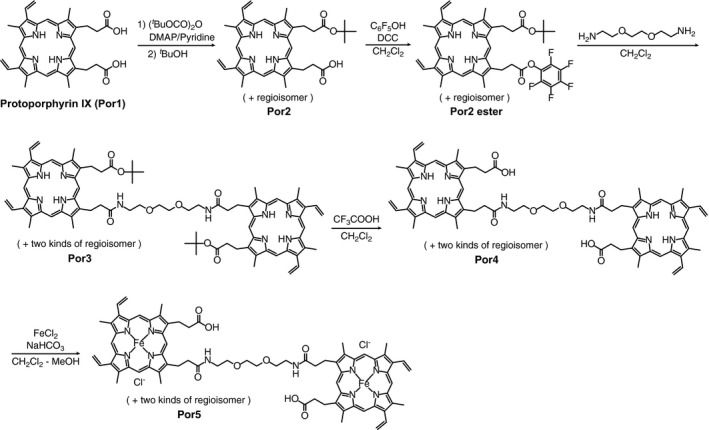
Procedure for the synthesis of the diheme compound from protoheme. The compounds were synthesized from protoporphyrin IX (Por1) according to Kitagishi *et al*. 22, as described in SI.

The diheme compound, Por5, was chemically synthesized from the protoheme and the linear diamine molecule, 1,8‐diamino‐3,6‐dioxaoctane, as shown in Fig. 2, according to the method described by Kitagishi *et al*. 22. The synthesized compounds were identified by UV–visible absorption, ESI‐TOF‐MS, 1H NMR spectroscopy, and reversed‐phase HPLC. The detailed data and experimental procedures for the diheme synthesis are described in Supporting Information.

### Diheme incorporation into 4HB

A solution containing 0.5–1.0 mm bHA in 25 mm Tris/HCl (pH 7.6) was mixed with Por5 dissolved in DMSO, either at molar ratio of 4HB and diheme of 1 : 1 or 2 : 1. The mixed solution was incubated at 4 °C for one month before the first AFM measurements (see Results and Discussion). The pH of the incubated solution was raised to 8.8, by adding 10 μL of 1 m Trizma base (pH 10.0) to 40 μL of the solution, and it was further incubated for 10 days at 4 °C. The resultant solution was used for the second AFM measurements.

### Spectrometry

UV–visible absorption spectra were recorded with either a Nano Drop ND‐1000 spectrophotometer (Thermo Scientific, Waltham, MA, USA) or a Hitachi U‐3000 spectrophotometer using a 1.0 mm path length quartz cuvette. CD spectra were recorded at 20 °C with a JASCO J700 spectropolarimeter using a 2.0 mm path length quartz cuvette. Mass analyses of the heme compounds and the polypeptide were conducted with a Bruker micrOTOF ESI‐QTOF mass spectrometer and a Shimadzu AXIMA MALDI‐TOF mass spectrometer using sinapinic acid as the matrix, respectively. The dynamic light scattering (DLS) measurements were performed with Dyna Pro 99‐E‐50 and MSTC‐800 instruments (Protein Solutions, Lakewood, NJ, USA) with a 12 μL microsample cell.

### AFM measurements

The diheme‐bHA samples (5 μL) were spotted onto a freshly cleaved, 5–10 mm^2^ mica plates. After a 3‐h incubation to adsorb the protein on the plate, the residual solution on the plate was removed by adding 200 μL of deionized water and wicking it away with a piece of filter paper placed at the edge of the plate. The sample plate was air‐dried at room temperature for a few days. AFM images were obtained with a Nano Scope IIIa (Digital Instruments, Tonawanda, NY, USA), using a phosphorus (n)‐doped Si scanning tip (Veeco Instruments, Plainview, NY, USA; spring constant 20–80 N/m, resonance frequency 245–289 kHz), with a scan rate of 0.5 Hz.

## Results and Discussion

**Figure 3:**
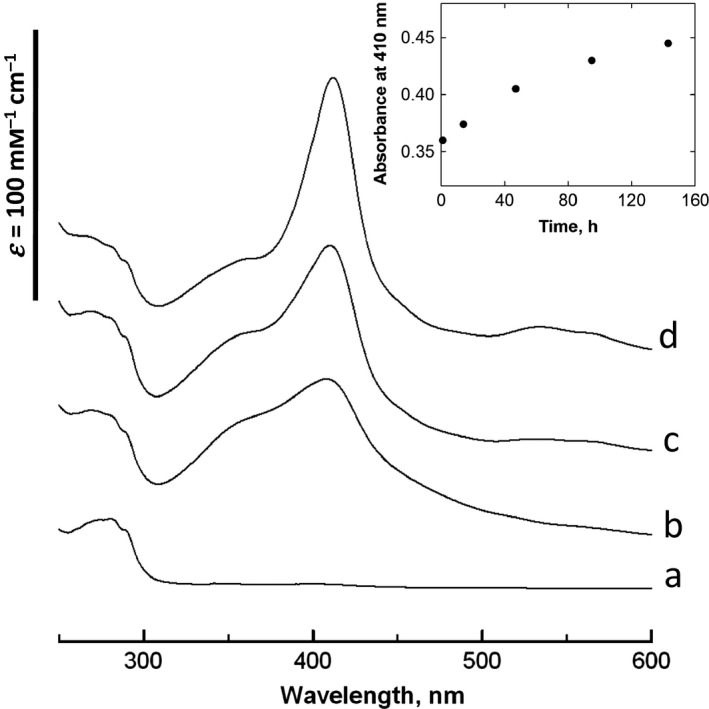
Changes in UV–VIS absorption spectra of bHA after Por5 addition. Absorption spectra of bHA without heme (a), incubated with Por5 for 2 h (b), for 6 days (c), and incubated with protoheme for 2 h (d). (Inset) Time course of the absorption at 410 nm of the mixture of Por5 and bHA. The protein was incubated with heme at the 2 : 1 molar ratio of 4HB and diheme, in a buffer solution containing 25 mm Tris/HCl (pH 7.6).

For *de novo* heme‐protein polymer formation, a protein solution, typically containing 0.5 mm bHA in 25 mm Tris/HCl (pH 7.6), was mixed with Por5 dissolved in DMSO, at a molar ratio of either 1 : 1 or 2 : 1 of 4HB and diheme. The changes in the UV–visible absorption spectrum indicated that the heme compound was gradually associated with the apo protein, through 6‐coordinate, bis‐histidine ligation of the heme iron, in a time range from days to a month (Fig. 3). The diheme was mixed with the protein in the ferric form, and no intermediate species such as the μ‐oxo‐complex of heme were detected during the polymer formation. The dynamic light scattering (DLS) measurements revealed that a fraction with hydrodynamic radii (*R*_h_) larger than 1 μm increased on a similar time scale (Table S1).

The Por5‐bHA assemblies were observed by tapping‐mode atomic force microscopy (AFM). For the AFM measurements, the protein solution mixed with Por5 was incubated at pH 7.6 for 30 days at 4 °C and then absorbed on a freshly cleaved 5–10 mm^2^ mica plate, as described in the Materials and methods. The sample plate was air‐dried in air at room temperature for a few days.

**Figure 4:**
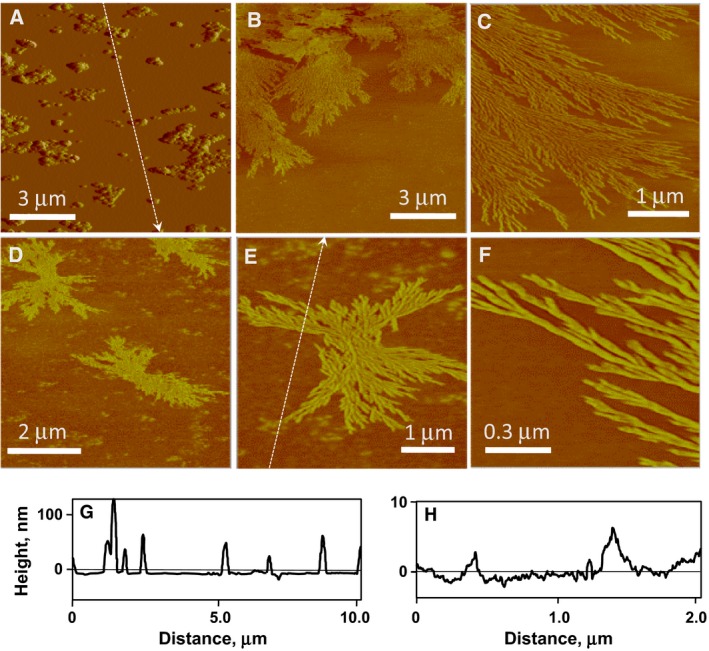
Atomic force microscopy images of Por5‐bHA assemblies. (A) The diheme compound was mixed with the 4HB protein at the molar ratio of 1 : 1 (Por5:bHA = 1 : 1) and incubated for 30 days at pH 7.6. (B) and (C) The same sample as in (A) was further incubated for 10 days at pH 8.8. (D)–(F) The diheme compound was mixed with the 4HB protein at the molar ratio of 2 : 1 (Por5:bHA = 2 : 1), incubated for 30 days at pH 7.6, and further incubated for 10 days at pH 8.8. (G) and (H) Section analyses of the AFM images in A and E, respectively, for the dashed lines.

The samples prepared as described above did not exhibit the expected fibrous materials, and instead, granular materials with diameters of 40–100 nm were observed in the AFM images (Fig. 4A,G). Then, pH of the heme‐protein solution was then raised to 8.8 by adding Trizma base, and it was further incubated for 10 days at 4 °C. During this incubation, DLS measurements indicated the increase in a fraction with an *R*_h_ of a few μm. The AFM images were collected, using the mica plates prepared with the resultant solutions, and fibrous materials were observed as shown in Fig. 4B–F. The heme‐protein assemblies formed highly organized, dendritic fibers with various lengths. Single fibers were branched and appeared to have submillimeter lengths along the fiber. The section analysis of the AFM images indicated that the vertical heights of the fibers were 1.0–5.0 nm, corresponding to approximate dimensions of the four or more helix bundles (Fig. 4H). These forms and lengths of the *de novo* heme‐protein assemblies were significantly different from those of the supramolecular polymers with native heme‐proteins reported previously 22, 23. This suggests that the usage of *de novo* proteins expands possibilities of the form and function of protein supramolecular assemblies.

The bHA protein used in the present study has the two heme‐binding sites in the 4HB unit composed of the bHA dimer. Thus, the branched fibers shown in the AFM images of Fig. 4B–F cannot be formed, if the association between the 4HB proteins occurs only through the linker chain between the ligated protohemes of Por5 (Fig. 1). Otherwise, the 4HB protein should be associated with other 4HB molecules through protein–protein interactions, creating junction points or nodes between linear fibers.

The granular materials observed in the AFM image of the sample prepared by the incubation at pH 7.6 (Fig. 4A) seem to be packed aggregates of the heme‐protein fiber observed in the higher pH sample. As the calculated isoelectric point of bHA is 7.8, the total net charge of bHA is close to zero at pH 7.6, and thus, it is prone to aggregate due to the low electric repulsion between the molecules. By increasing the sample pH, the net charge of the molecule becomes negative, and the fiber aggregates may be unraveled to form the branched fibers, as shown in Fig. 4B–F.

The time range of the polymer formation from days to a month (Fig. 3 and Table S1) was much longer than those of binding single heme to the 4HB and many native apo heme‐proteins. The heme‐binding to apo heme‐proteins contains at least two reactions. One is the diffusion process of heme and protein molecules, including rotation, and the other is the heme ligation. In the case of diheme binding, one of the linked hemes binds to an apo heme‐protein at first, in a similar manner to the single heme‐binding. However, the second heme‐binding contains binding of the nonligated heme associated with the holo protein to another apo heme‐protein. The latter reaction is expected to be much slower than the former and is rate‐limiting. Furthermore, the *de novo* heme‐protein fiber displayed highly ordered dendritic forms, and thus, the polymer formation may involve large conformational entropy decrease 27. This may also be attributable to the slower polymer formation.

In conclusion, we have designed and prepared highly organized redox protein polymers, in which monomer–monomer interactions occur through a newly synthesized diheme compound and its ligation into the *de novo* heme‐protein. The polymer was formed to be branched, dendritic fibers, possibly through interactions between proteins as well as those between protein and heme. The 4HB scaffold used here can bind four hemes using bHH (see Fig. 1), which may enable modulation of the polymer form, *for example*, branching frequency. *De novo* heme‐proteins are promising molecules for electron transfer, ligand binding, and enzymatic reactions beyond the natural protein functions 6, 7, 8, 9, 10, 11, 12. Thus, the present approach with a *de novo* heme‐protein and a diheme compound will yield functional mesoscopic devices with electronic and other functions.

## Author contributions

YI conceived and designed the project. RN and SK synthesized the heme compounds. YI synthesized the protein and protein polymer. ET and MK performed the AFM measurements and the data analysis. YI wrote the study.

## Supporting information


**Fig. S1.** Reversed‐phase HPLC chromatogram of Por5.
**Fig. S2.** ESI‐TOF‐MS spectrum of Por5.
**Fig. S3.** UV‐VIS absorption spectra of protoporphyrin IX (Por1), Por4, hemin and Por5.
**Table S1.** Measurements of the *de novo* heme‐protein polymer formation by DLS.
