# Supplementary material for: Supramolecular polymer formation by a de novo hemoprotein with a synthetic diheme compound
Source: FEBS Open Bio. 2018 May 11;8(6):940–6. doi: 10.1002/2211-5463.12424 (PMC5986056; doi:10.1002/2211-5463.12424)
Supplement: Supplementary file 1 — Fig. S1. Reversed‐phase HPLC chromatogram of Por5. Fig. S2. ESI‐TOF‐MS spectrum of Por5. Fig. S3. UV‐VIS absorption spectra of protoporphyrin IX (Por1), Por4, hemin and Por5. Table S1. Measurements of the de novo heme‐protein polymer formation by DLS. [file FEB4-8-940-s001.docx]

**Supplementary Information for**

"Supramolecular polymer formation by *de novo* hemoprotein with a synthetic diheme compound" by Y. Isogai et al. 2018.

**Preparation of diheme compound Por5**

The synthetic details of **Por5** are described below, according to the method described by Kitagishi et al. [S1]. The chemical structures are shown in Fig. 2 of the main text.

**Por2:** Protoporphyrin IX mono t-butyl ester (**Por2**) was synthesized from protoporphyrin IX (**Por1**) by the same procedures reported in the literature [S2].

**Por2 ester:** In a 50 mL round bottom flask under Ar atmosphere, the protoporphyrin IX mono t-butyl ester (**Por2**) (200 mg, 0.32 mmol), pentafluorophenol (70 mg, 0.38 mmol) and dicyclohexylcarbodiimide:DCC (78 mg, 0.38 mmol) were dissolved in 20 mL of dichloromethane. The solution was stirred overnight at room temperature in the dark. After the extent of condensation was checked by TLC, the solvent was evaporated. The residue was passed through a short column chromatography (SiO_2_, CH_2_Cl_2_:diethylether = 19:1, v/v). The eluent was collected and concentrated to give crude **Por2 ester**　(114 mg, 45%), and this was used without further purification. ^1^H NMR (400MHz, CDCl_3_) δ 10.18 (s, 1H) 10.14 (s, 1H) 10.07 (s, 1H) 10.05 (s, 1H) 8.31-8.22 (m, 2H) 6.41-6.34 (m, 2H) 6.22-6.16 (m, 2H) 4.49 (t, J = 7.8 Hz, 2H) 4.34 (t, J = 7.6 Hz, 2H) 3.72-3.60 (m, 14H) 3.15 (t, J = 7.8 Hz, 2H) 1.33 (s, 9H) {NH protons are broadening and not determined.}; ESI-TOF-MS (positive mode) m/z 785.34 [M + H]^+^, calcd. for C_44_H_42_F_5_N_4_O_4_ 785.31

**Por3:** In a 50 mL round bottom flask under Ar atmosphere, **Por2 ester** (100 mg, 0.127 mmol) and 1,8-diamino-3,6-dioxaoctane (30 mg, 0.20 mmol) were dissolved in 50 mL of dichloromethane. The solution was stirred and refluxed overnight in the dark. The solvent was evaporated, and the residue was subjected to column chromatography (SiO_2_, CH_2_Cl_2_:acetone = 1:1, v/v). After the first fraction collected and concentrated, the residue was recrystallized from dichloromethane-hexane. The purple solid was dried to obtain crude **Por3** (47 mg, 54%). ^1^H NMR (400MHz, CDCl_3_) δ 9.58-9.08 (16 singlet peaks, 8H) {the signals of the meso-protons split unseparated 16 signals due to the presence of 3 kinds of regioisomer: A-A, A-B, B-B.} 8.06-7.76 (m, 4H) 6.59-6.52 (m, 2H) 6.24-5.98 (m, 8H) 4.03-4.01 (m, 8H) 3.97-3.64 (m, 8H) 3.55-3.00 (m, 28H) 2.90-2.70 (m. 8H) 1.57 (s, 18H) {NH protons are broadening and not determined.} The signals between 4.03 to 2.70 could not be clearly identified due to the presence of regioisomers and by-product. The latter was able to be separated at next reaction.; ESI-TOF-MS (positive mode) m/z 1349.64 [M + H]^+^, calcd. for C_82_H_97_N_10_O_8_ 1349.75

**Por4:** In a 50 mL round bottom flask under Ar atmosphere, **Por3** (60 mg, 0.044 mmol) was dissolved in 10 mL of dichloromethane. The solution was cooled in an ice bath. To the solution was added 4 mL of trifluoroacetic acid. The solution was stirred for 7 h at room temperature in the dark. After the solution was evaporated, the residue was recrystallized from methanol-diethylether. The precipitate was dried to give **Por4** (30 mg, 55%). ^1^H NMR (400MHz, DMSO-d_6_) δ 12.35-12.30 (br, 2H) 9.81-9.54 (m, 8H) 8.30-8.08 (m, 4H) 7.83-7.82 (m, 2H) 6.34-6.24 (m, 4H) 6.19-6.06 (m, 4H) 4.12-4.11 (m, 4H) 3.98-3.94 (m, 4H) 3.51-3.21 (m, 28H) 3.08-3.05 (m, 4H) 3.03-3.00 (m, 4H) 2.90-2.87 (m, 8H) {NH protons are broadening and not determined.}; ESI-TOF-MS (positive mode) m/z 1259.58 [M + Na]^+^, calcd. for C_74_H_80_N_10_NaO_8_ 1259.61

**Por5:** In a 50 mL round bottom flask, **Por4** (40 mg, 0.032 mmol), ferrous chloride tetrahydrate (290 mg, 1.46 mmol) and NaHCO_3_ (40 mg, 0.047 mmol) were dissolved in 16 mL of dichloromethane and 3.5 mL of methanol. The reaction mixture was stirred for 4h at 40 °C in the dark. After the solvent was evaporated, the residue was dissolved in dichloromethane-methanol. The solution was washed with 0.05 M HCl and distilled water. The organic layer was dried over Na_2_SO_4_. After the solvent was evaporated, the residue was recrystallized from dichloromethane (3.3 mL)/methanol (1.7mL)-diethylether (20 mL). The precipitate was dried to give crude **Por5** (28 mg, 62%). This was further purified by reversed-phase HPLC (Figure S1). ESI-TOF-MS (negative mode) m/z 1413.38 [M - H]^-^, calcd. for C_74_H_75_Cl_2_Fe_2_N_10_O_8_ 1413.66; ESI-TOF-MS (positive mode) m/z 672.22 [M - 2Cl]^2+^, calcd. for C_74_H_76_Fe_2_N_10_O_8_ 1344.45 (Figure S2).


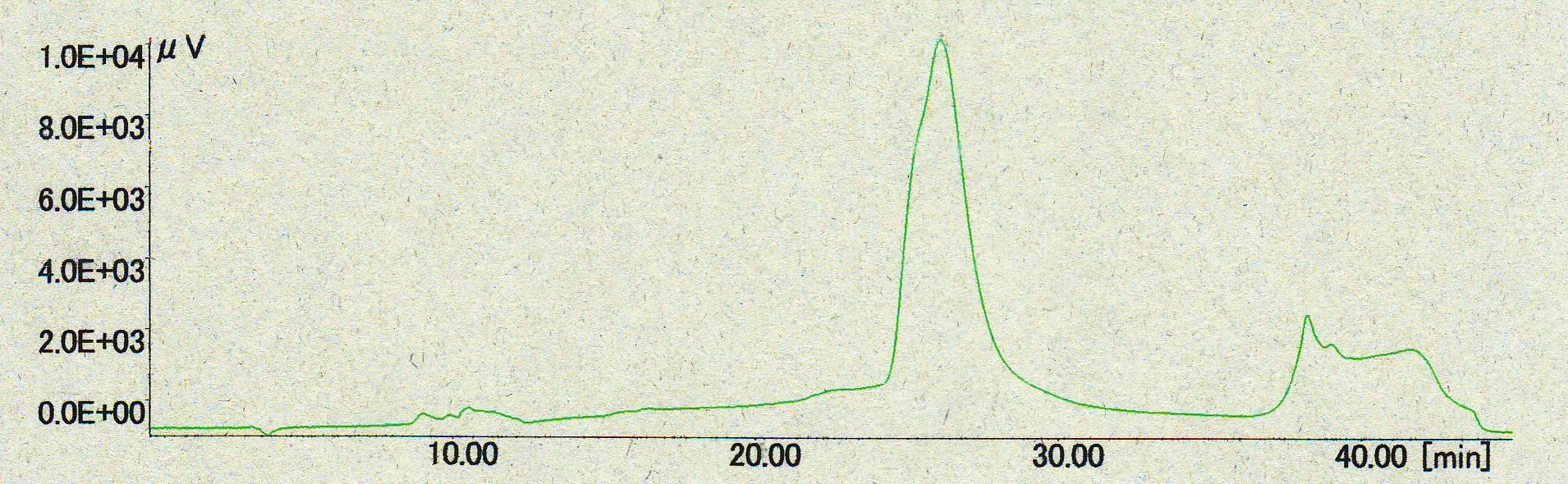


Por5

**Figure S1.** Reversed-phase HPLC chromatogram of Por5. The analysis was performed using a COSMOSIL 5C18-AR packed column (4.6×250 mm, Nacalai Tesque) with a 40 to 70% acetonitrile gradient in 0.05% trifluoroacetic acid (TFA), at a flow rate of 1.0 ml/min. The chromatogram was monitored at 400 nm with a Hitachi L-6200 HPLC system. The broad peak may be due to the flexible structure of Por5.

**
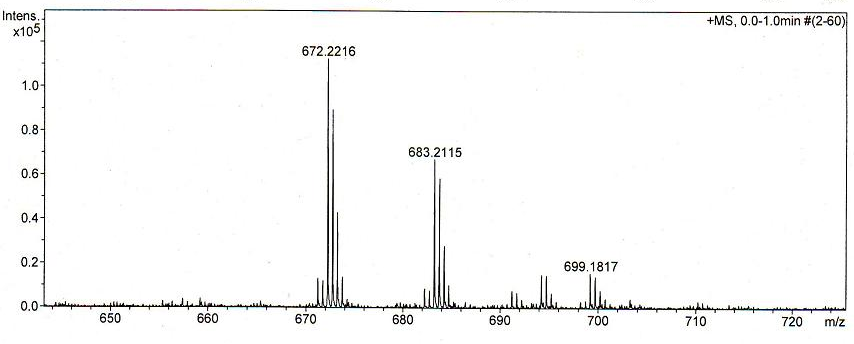
**

**Figure S2.** ESI-TOF-MS (positive mode) spectrum of the synthetic diheme compound, Por5. Mass calculated for [C_74_H_76_Fe_2_N_10_O_8_]^2+^ = 1344.45, *m/z* = 672.22.


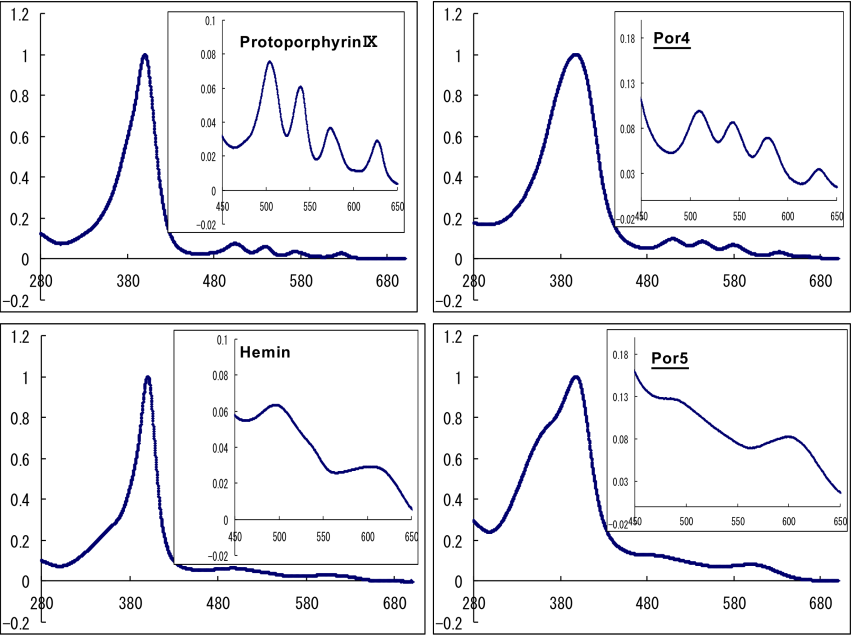


**Figure S3.** UV-VIS absorption spectra of protoporphyrin IX (Por1), Por4, hemin and the diheme compound Por5. The solvents are CHCl_3_/Methanol (2:1, v/v). These chemical structures are shown in Fig. 2 of the main text. The spectra of Por4 and Por5 in the wavelength range between 450 and 650 nm are characteristic of porphyrin and heme, respectively.

**Table S1.**  Measurements of the *de novo*

heme-protein

polymer formation by DLS.

| Time (day) | Radius（nm） | Intensity （%） | Mass (%) |
| --- | --- | --- | --- |
| 0 | 76.2 | 96.4 | 100 |
|  | >1000 | 3.6 | 0.0 |
| 7 | 118.7 | 92.3 | 99.9 |
|  | >1000 | 7.7 | 0.1 |
| 16 | 68.9 | 55.1 | 99.8 |
|  | >1000 | 44.9 | 0.2 |
| 21 | 78.0 | 50.5 | 99.7 |
|  | >1000 | 49.5 | 0.3 |

The measurements were performed after incubation of the protein solution at a molar ratio of 4HB and diheme of 1:1 in 25 mM Tris-HCl (pH 7.6).

**SI References**

[S1] Kitagishi, H., Oohora, K., Yamaguchi, H., Sato, H., Matsuo, T., Harada, A., Hayashi, T. (2007) Supramolecular hemoprotein linear assembly by successive interprotein heme-heme pocket interactions. *J. Am. Chem. Soc.* 129, 10326-10327.

[S2] Matsuo, T; Hayashi, T; Hisaeda, Y. (2002) Reductive activation of dioxygen by a Myogrobin reconstituted with a flavohemin. *J. Am. Chem. Soc.* 124, 11234-11235.
